# Professional quality of life and organizational changes: a five-year observational study in Primary Care

**DOI:** 10.1186/1472-6963-7-101

**Published:** 2007-07-04

**Authors:** Jesus Martin-Fernandez, Tomas Gomez-Gascon, Milagros Beamud-Lagos, Jose Alfonso Cortes-Rubio, Angel Alberquilla-Menendez-Asenjo

**Affiliations:** 1 San Martin de Valdeiglesias Health Centre, 8^th ^Primary Care Area, Madrid, Spain; 2 Puerta Bonita II Health Centre, 11^th ^Primary Care Area, Madrid, Spain; 3 Research Unit, 11^th ^Primary Care Area, Madrid, Spain; 4 Ministry of Health and Consumer Affairs, Madrid, Spain; 5 Information System, 11^th ^Primary Care Area, Madrid, Spain

## Abstract

**Background:**

The satisfaction and the quality of life perceived by professionals have implications for the performance of health organizations. We have assessed the variations in professional quality of life (PQL) and their explanatory factors during a services management decentralization process.

**Methods:**

It was designed as a longitudinal analytical observational study in a Health Area in Madrid, Spain. Three surveys were sent out during an ongoing management decentralization process between 2001 and 2005. The professionals surveyed were divided into three groups: Group I (97.3% physicians), group II (92.5% nurses) and group III (auxiliary personnel). Analysis of the tendency and elaboration of an explanatory multivariate model was made. The PQL -35 questionnaire, based on Karasek's demand-control theory, was used to measure PQL. This questionnaire recognizes three PQL dimensions: management support (MS), workload (WL) and intrinsic motivation (IM).

**Results:**

1444 responses were analyzed. PQL increased 0.16 (CI 95% 0.04 – 0.28) points in each survey. Group II presents over time a higher PQL score than group I of 0.38 (IC 95% 0.18 – 0.59) points. There is no difference between groups I and III.

For each point that MS increases, PQL increases between 0.44 and 0.59 points. PQL decreases an average of between 0.35 and 0.49 point, for each point that WL increases.

Age appears to have a marginal association with PQL (CI 95% 0.00 – 0.02), as it occurs with being single or not having a stable relationship (CI 95% 0.01 – 0.41). Performing management tasks currently or in the past is related to poorer PQL perception (CI 95% -0.45 – -0.06), and the same occurs with working other than morning shifts (CI 95% -0.03 – -0.40 points).

PQL is not related to sex, location of the centre (rural/urban), time spent working in the organization or contractual situation.

**Conclusion:**

With the improvement in work control and avoiding increases in workloads, PQL perception can be maintained despite deep organizational changes at the macro-management level. Different professional groups experience different perceptions depending on how the changes impact their position in the organization.

## Background

From the perspective of organizational development change is a set of values, strategies and techniques for the purpose of enhancing individual development and improving organizational performance. Organizational changes are divided into first and second-order changes. First-order change is a normal operational change (i.e. working hours, shifts). Second-order change concerns changes in the system itself (i.e. a new financial system, a new work scenario, etc.). Other contemporary theories include the distinction between change that is episodic and intermittent and change that is continuous and incremental. 'Episodic change' is infrequent, discontinuous and intentional and occurs at a macro-level. 'Continuous change' is often recurrent, cumulative, emergent and self-organizing and occurs at the micro-level [1].

A changing environment characterizes health care systems. The rapid evolution of medical technology and social expectancy is associated with increasing job complexity and relates to a changing work environment. Health care is a growing employment sector in advanced societies, and quality of work among health care professionals varies widely. Changing work conditions in health care systems has shown a negative impact on psychological wellbeing in healthcare workers, generating job stress and dissatisfaction [2-4].

Two models are especially accepted in occupational stress research, the effort-reward imbalance (ERI), and the demand-control model. The effort-reward imbalance (ERI) model focuses on a negative trade-off between experienced 'costs' and 'gains' at work [5]. In the demand-control model work conditions characterized by low control and high demand have been related to poor psychological wellbeing. This model places its emphasis on the distinct characteristics of the work place: job task profiles defined by high quantitative demands and a low degree of decision latitude or task control are assumed to elicit sustained stress reactions [6]. Both models have successfully predicted the emergence of health outcomes associated to a low imbalance between efforts and rewards [7] or to a low control/high demand work environment [8].

The definition of job satisfaction is made in contraposition to the concept of job stress, which, according to the demand-control model, represents a disequilibrium perceived between the demand and the individual's capacity for response, in conditions where failure in the face of this demand can have important consequences [9]. Job satisfaction in the health organization setting, in competitive ambits, appears to be a guarantee of the maintenance of human capital [10,11], and has been shown to improve the quality of professional practice, and the perception the patient has of it. In addition, the reduction of professional satisfaction had a negative impact on the health care organization performance [12,13]. So, there is an important ongoing debate about the state of professional satisfaction among health care givers and its consequences on the impact this can have on health care organizations [14].

In our country we have recently undergone great organizational change that could be classified as a "second-order" "episodic" change at the macro management level and could affect the perception of professional wellbeing. In Spain, the public health system is organized as a national health system with management responsibilities that are very decentralized in the regional governments and that is fully financed by taxes and articulated at two organizational levels: primary care and hospital-based care. All Spanish citizens and foreigners who meet the legal requirements are entitled to care. The rendering of services embraces all types of pathologies except some dental health treatment and beauty care problems. Primary care is the entry level of health care. Primary care acts as entry door to the system, as case and coordinator manager and as flow regulator, which guarantees the globality and continuity of care throughout the patient's life. Intervention at this level includes health maintenance and recuperation activities and others in health promotion, health education and disease prevention [15]. These services were managed by the central government until 20 years ago when the decentralization process gradually began in all the country. This did not take place in our region until 2002. Following the publication the Health Organization Law (Ley de Ordenación Sanitaria) of the Community of Madrid (LOSCAM), a new scenario is set in place that turns on decentralization, deconcentration, autonomy and responsibility for the management of services [16]. Decentralization represents a substantial turning point in the form of funding services, which now depend on the taxes collected by the each regional government.

Changes were expected once the transfer of services management powers to regional governments was completed. Regulation of job conditions and the offering of services were expected to change in two stages, one by homogenization and the other by improvement [17]. The improvement of the services portfolio and the population increase have been quicker than the adaptation of investment, which has influenced the perception of a greater workload by health professionals, specifically in the organization of primary care. On the one hand, there has been a certain competition in health supply between regional governments, which has tended to increase the services offered [18]. This has been accompanied by a significant increase in the population susceptible to utilize these services. This population has especially increased due to migratory flows of an economic character. In our community the population increased from 5,372,000 to 5,964,000 persons in the 2001–2005 period without a parallel increase in health professionals in this same period.

However, until 2005 professionals did not perceive significant changes in compensation systems or in labour relations with the financer, or in relation to other care levels or in the professional activity evaluation systems.

Other "second-order," "episodic" changes in national health systems in our setting produced a significant variation in professional quality of life perception [3,4,19]. We believed it relevant to monitor the PQL in a Primary Care Health Area measured by a specific questionnaire constructed under the demand-control model, and to study the explanatory factors and their stability during the time period that embraced all changes in the management model.

## Methods

The population studied was professionals assigned to Primary Care Area 11, which includes 1,500 workers and covers more than 790,000 patients.

Professionals were divided into three homogeneous subgroups according to grade and salaries received. Group I included mainly physicians, some pharmacists, dentists and psychologists. Group II consisted of nurses, midwives, physiotherapists and social workers and group III was made up of auxiliary office workers, hospital porters and clinical auxiliaries. All fulfilled their jobs in health centres. These groups correspond to administrative categories A (group I), B (group II) and C, D and E (group III). A validated questionnaire in Spanish, PQL-35, was handed out at three different times in 2001, 2003 and 2005. On the first two occasions, questionnaire was sent to a stratified random sample of 450 workers, 150 subjects in each professional subgroup (I, II, III). In 2005 the same questionnaire was administered to all the population of the study (1,449 subjects). An anonymous and free system of return was used for the survey. In the analysis of the first survey we found four answers from the Management Area Centre, in which professionals had no direct contact with patients. These replies were excluded and in the following years these professionals did not receive surveys (in 2003 they were excluded from the randomization list and in 2005 they did not receive the questionnaires).

The instrument for measuring PQL was questionnaire PQL-35. This questionnaire was constructed based on the demand-control model formulated by Karasek and has been validated in the Spanish language [9,20]. It consists of 35 questions that are answered on a scale from 1 to 10, which apply the categories "nothing/none" (values 1 and 2), "some" (values 3, 4 and 5), "sufficient/enough" (values 6, 7 and 8) and "many/much" (values 9 and 10). The PQL-35 presents three major dimensions: "management support," "workload" and "intrinsic motivation," in addition to a direct question on PQL perception. Each dimension is constructed from a linear combination of test answers. "Workload" domain (12 items) relates to the demand component of Karasek's model. "Management support" (11 items) and "intrinsic motivation" (9 items) compose the resources for coping with the demands (control component of the model). The outcome measurement of PQL is the answer to a direct question ("I feel my professional quality of life...") on the mentioned scale. There are two other questions, one that does not correspond to any domain, and another only used for professionals with management responsibilities.

This test has been widely used in our setting to evaluate PQL [21], to identify areas for improvement [22] and for exploring the effects of organizational climate on health care workers [23].

Other independent variables were also collected. These included age, gender, type of family relationship (stable couple/others), professional group, employment situation (indefinite contract/with limited duration), working hours (mornings only/others), type of centre (urban/rural), average care pressure in categories (high/low, more than 45 patients/day for group I and 25 patients/day for II; group III does not have directly assigned patients), current or past performance of management work and the time-length working in the Health Area. Independent variables such as scores obtained were also considered in each of the three sub-scales of the questionnaire: management support, workload and intrinsic motivation. The variable in the result was the PQL measurement obtained by a direct question in the PQL-35.

Graphing methods compare measurements at each point of study and the averages were compared with an ANOVA tendency test or with non-parametric methods (Kruskal-Wallis and Jonckheere-Terpstra tests).

For the study of the variables associated with professional quality of life an explicative multiple linear regression model was constructed. The variable "year of the study" was codified in three ordered categories (1, 2 and 3) and was included first of all in the model. The variable "professional category," was codified as two "dummy" variables that compared groups II and III with group I. A backward-steps method was utilized to obtain the most parsimonious model [24]. Residuals analysis assured that the assumptions of the model of normality, linearity, independence and homogeneity of the variances were respected.

Analysis was done using the SPSS 13.0 programme (Licence no. 9852553).

## Results

We analyzed 1,444 questionnaires, with a response rate of 61.1, 64.0 and 60.8%, respectively, for the years 2001, 2003 and 2005.

Table 1 shows the characteristics of the subjects studied in each year; 97.3% of the subjects of group I were family physicians and paediatricians, 92.5% of group II nurses and 84.2% of group III office worker auxiliaries and porters. This distribution is a fairly approximate reflection of the composition of the population surveyed.

**Table 1:** Characteristics of participants in study

	2001	2003	2005
Age in years ^a^	41.6 (40.4–42.8)	42.2 (40.7–43.6)	44.7 (44.0–45.3)
Female sex ^b^	77.9 (72.8–83.0%)	70.8 (65.4–73.2%)	76.8 (74.0–79.6%)
Lives with partner ^b^	74.0 (68.2–79.8 %)	74.9 (69.4–80.4%)	64.7 (61.5–67.9%)
Group I ^b^	32.3 (26.5–38.1%)	37.4 (31.4–43.4%)	42.9 (39.6–46.2%)
Group II ^b^	40.9 (34.9–46.9%)	30.3 (24.6–36.0%)	36.7 (33.5–39.9%)
Group III ^b^	26.8 (21.4–32.2%)	32.3 (26.5–38.1%)	20.4 (17.7–23.1%)
Indefinite contract ^b^	51.5 (45.4–57.6%)	55.4 (49.6–61.2%)	47.0 (43.7–50.3%)
Urban team ^b^	89.2 (85.4–93.0%)	90.1 (86.6–93.6%)	88.1 (79.1–84.3%)
Management responsibilities ^b^	34.2 (28.1 40.3%)	34.2 (28.1 40.3%)	32.4 (29.3–35.5%)
Morning shift ^b^	53.5 (47.4–59.6%)	56.2 (50.4–62.0%)	48.4 (45.1–51.7%)
Years in Area ^a^	10.4 (9.5–11.3)	11.3 (10.5–12.1)	11.2 (10.8–11.7)
High care pressure ^b^	37.3 (29.8–44.8%)	29.8 (23.1–36.5%)	53.7 (50.0–57.4%)

^a ^Average, in parenthesis the Confidence Interval of 95%

^b ^Percentage over total responses, between parenthesis Confidence Interval of 95%.

**Figure 1:**
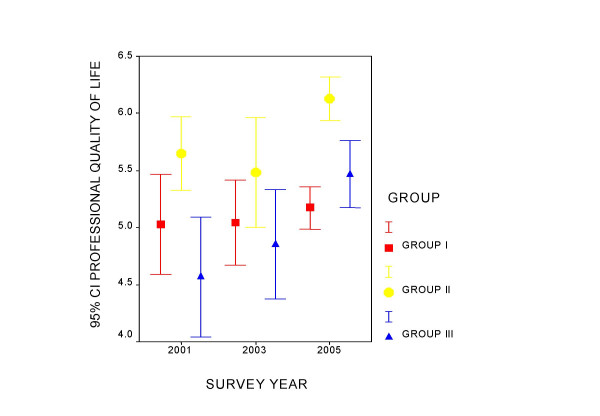
Evolution of the average value of the perception of the professional quality of life by professional category.

**Figure 2:**
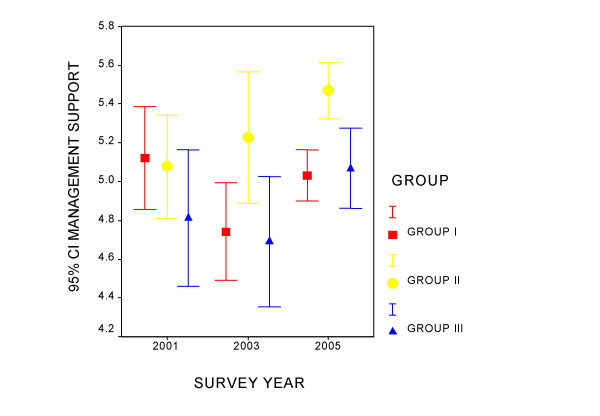
Evolution of the management support component by professional category.

**Figure 3:**
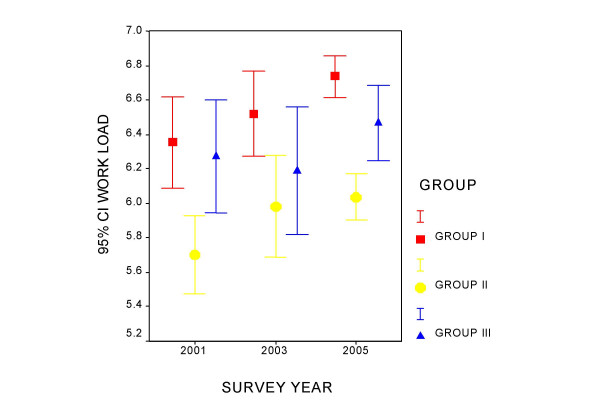
Evolution of the workload component by professional category.

**Figure 4:**
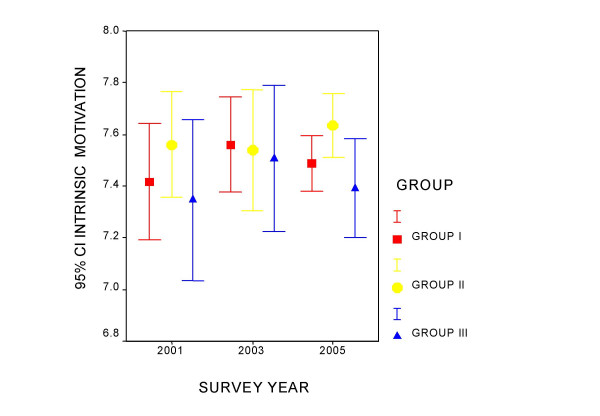
Evolution of the intrinsic motivation component by professional category.

Figures 1 to 4 present the measurement of quality of life perception and of the three components of the PQL -35 throughout the study period, for each professional category. Group I maintains a constant quality of life perception throughout of around value 5, and denotes an increase in the perception of the workloads that differentiates it from the other two groups. In group II a tendency is observed in the time of improvement of the evaluation of PQL and greater management support, as well as a slight increase in workloads. Group III appears to improve quality of life and the perception of management support over the course of time. Intrinsic motivation remains stable for all the groups during the period studied.

Table 2 presents the ANOVA for tendencies for each component in each professional category. Group I shows a tendency towards a perception of greater work loads in the successive mailings, which is significant. Group II improves the perception of PQL. Management support and the perception of workloads also increase in this group. In the case of PQL in group II the variances were observed not to be homogeneous and the analysis was repeated using Kruskal-Wallis and Jonckheere-Terpstra tests, both showing signification (p = 0.006 and p = 0.002, respectively).

**Table 2:** Analysis of tendencies for each domain in each professional category.

		2001^a^	2003^a^	2005^a^	P^b^
GROUP I	PQL	4.59–5.46	4.67–5.41	4.99–5.36	0.436
	Management support	4.82–5.36	4.49–5.00	4.89–5.10	0.709
	Workload	6.14–6.67	6.29–6.78	6.61–6.86	0.015
	Intrinsic motivation	7.16–7.62	7.38–7.75	7.38–7.59	0.669
GROUP II	PQL	5.32–5.97	5.00–5.96	5.94–6.32	0.004
	Management support	4.81–5.37	4.79–5.48	5.32–5.61	0.009
	Workload	5.46–5.93	5.73–6.34	5.90–6.17	0.026
	Intrinsic motivation	7.34–7.77	7.25–7.73	7.52–7.77	0.389
GROUP III	PQL	4.04–5.09	4.37–5.33	5.17–5.77	0.001
	Management support	4.43–5.15	4.32–5.03	4.88–5.29	0.076
	Workload	5.94–6.62	5.85–6.55	6.24–6.68	0.275
	Intrinsic motivation	6.99–7.63	7.20–7.78	7.21–7.59	0.757

^a ^The 95% Confidence Interval is presented for the average in each year of study.

^b ^Value of the signification for the linear component of a tendency test (ANOVA) PQL: professional quality of life.

Throughout the observation period Group III improves PQL perception and the tendency towards the perception of greater management support remains at the edge of signification.

**Figure 5:**
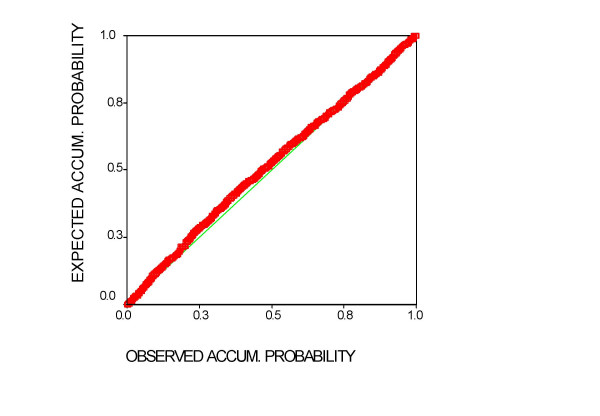
**P-P normal graph of standardized residuals regression of the model chosen**. In a perfect normal distribution all values would be on the diagonal (observed and expected probabilities would coincide).

Table 3 reflects the summary of the multivariate model chosen, which explains the 35.8 of the variance. The analysis of the tolerance reasonably discards the existence of colinearity. The Durbin Watson statistic (d = 2.01) indicates that the assumption of independence is respected. Figure 5 shows that the residuals are normally distributed, a prior condition for evaluating this type of model.

**Table 3:** Coefficients of the explanatory model

				Confidence interval of 95%		Colinearity	
Variables	Beta	T	Sig.	Lower limit	Upper limit	Tolerance	VIF

Constant	2.40	4.35	0.00	1.32	3.48		
Group II vs I	0.38	3.71	0.00	0.18	0.59	0.77	1.29
Group III vs I	0.00	-0.02	0.99	-0.24	0.23	0.82	1.23
Survey year	0.16	2.53	0.01	0.04	0.28	0.92	1.08
Management support	0.52	13.25	<0.01	0.44	0.59	0.70	1.43
Work load	-0.42	-11.52	<0.01	-0.49	-0.35	0.89	1.13
Intrinsic motivation	0.24	5.07	<0.01	0.15	0.33	0.74	1.34
Age	0.01	1.71	0.09	0.00	0.02	0.84	1.20
Others vs. Stable relationship	0.21	2.09	0.04	0.01	0.41	0.92	1.08
No management tasks vs. management tasks	0.25	2.59	0.01	0.06	0.45	0.91	1.10
Others vs. Morning shift	-0.21	-2.25	0.02	-0.40	-0.03	0.86	1.16

Dependent variable: Professional quality of life

VIF: Variance Inflation Factor

From the analysis of the independent variables coefficients, in relation to the comparison between professional groups, group II clearly presents better PQL perception than group I of between 0.18 and 0.59 points. There are no relevant differences between groups III and I. A tendency towards improvement in quality of life is intuited, with an average increase of between 0.04 and 0.28 points in each successive survey.

Of the three domains into which the PQL 35 is grouped those that have greater weight in quality of life are management support and workload. For each point that management support increases, quality of life increases an average of between 0.44 and 0.59 points, while decreasing an average of between 0.35 and 0.49 points when workloads increase.

With respect to personal characteristics worker age appears to have a marginal association with PQL, increasing by an average of between 0 and 0.2 points per decade (p = 0.09). Being single or not having a stable relationship is also associated with better quality of life perception of between 0.01 and 0.41 points.

Having performed management tasks currently or in the past is related to poorer perception of between 0.06 and 0.45 PQL points, and the same occurs with having worked shifts other than morning (-0.21; CI 95% -0.03 – -0.40 points).

In this model PQL is not related to sex, location of the centre (rural/urban), time spent working in the organization or contractual situation.

## Discussion

PQL perception is an important variable in the study of organizational changes. In our setting a "second-order," "episodic" organizational change has not represented a decrease in PQL. On the contrary, its variation over the course of time adopts an ascending direction, although the importance of this change is not well defined.

The impact of the changes that our health system is undergoing has different repercussions in the different professional groups and in the different dimensions of the PQL concept.

Variations of groups II (fundamentally composed of nurses) and III (auxiliary personnel) are clearly positive, as they sense an improvement in resources, principally those linked to the organization ("management support"). So it is not surprising that PQL improves in both groups.

Group I (93% physicians) is characterized by an increase in the demands component in this period. Group II also perceives this change although it has a better basal situation than the other two groups.

We confirmed the influence of the "work load" demands in Karasek's model on PQL perception. This has been previously shown in cross-sectional studies using the PQL-35 questionnaire [9,22,23]. The other part of the model, work control, is a fundamental component of the stress process [8]. The PQL-35 questionnaire shows two domains that could be grouped in the Karasek model "control" component: "management support" and "intrinsic motivation." The weight of "management support" on PQL perception was proven over the time. The role of "intrinsic motivation" is less clear. In some studies "intrinsic motivation" was related to greater stress [25], but in others motivation along with a good acceptance of changes were related to better job satisfaction [2]. As expected, we found "intrinsic motivation" to be a consistent resource to copy changes over time.

Nevertheless, we found some notable results in a particular group. Professionals with management responsibilities (heads of primary care teams) perceived a poorer PQL. This is relevant as they are at the top of task-level control in the organization. The concept of work-control has been of central importance in theoretical formulations in job stress. We understand that improving "work control" (measured as an increase in "management support") could be related to better quality of life perceptions in groups II and III. So we were surprised that workers with a higher "control" in the organization, as "directors" in primary care teams, perceived poorer PQL. This may respond to the absence of leadership and of a developmental model in the top management of the health system. Although we had previously found no difference with respect to the perception of management support in this group [22], this would be an interesting aspect to study to improve the management of human capital in the organization.

There is in the recent literature a reflection on the worsening in satisfaction of primary care professionals [14], mainly physicians, which we have not observed. This dissatisfaction, detected even in professionals in training, has been attributed to the lack of fulfilment of expectations and to a lack of control over the setting [26]. Professionals in our setting may share these two aspects, there being many studies that show this [21-23,27], but a worsening tendency is not confirmed, at least in the last five years. The differences with other processes of change in other health organizations may stem from experiencing change as an opportunity for improvement [17], and that the change in the organization has not reached micromanagement [18]. If changes are perceived by professionals as challenging, some authors have found them associated positively with perceptions of job satisfaction and stress [2]. Moreover, professionals, though they have perceived a workload increase, have not seen their autonomy in professional decisions limited. Neither have there been significant changes in the evaluation of their work, nor in the salary perception systems until the last year evaluated. These factors, together with a perception of sufficient management support, seem to have prevented the deterioration in PQL perception [4,19,28].

We have collected other characteristics that could be related to different PQL perceptions, because they could modify the perception of demands or work control.

The role of age in relation to satisfaction is controversial. In some studies it seems to protect against job stress [25], while in others this increases over time in practice [27]. Perhaps there are other factors, not measured in this study, which could increase with time and could improve job control, such as the position in the team and social support.

Working on shifts less adapted to family needs (with care-giving outside regular working hours or night shifts) is shown to be less desirable for the professional, as has been mentioned [2,29,30]. This is congruent with the fact that persons who live according to relationship models other than the stable couple have fewer adaptability problems and perceive better PQL.

We consider that these characteristics could be divided into those that increase demands (working shifts) and those that relate to a better capacity to copy organizational changes, and in the end to better work control and better PQL perception (age, living alone, etc.).

This paper confirms the role of demands and control in PQL perceptions, and its stable significance over time. Moreover, it reveals that some characteristics of the subject and the organization also fit this model.

Although PQL/job satisfaction analysis is based on a solid conceptual framework, the Karasek demand-control model using a previously validated tool, possible weaknesses in the study need to be discussed. The sensitivity to change of the instrument still has not been determined, which does not allow us to evaluate the findings on the professional's perception in all their extension.

This being a "populational" study, we are not in a position to affirm that the variables studied do not weigh on individual PQL perception, nor can interventions on them at the individual level be recommended. Moreover, it cannot be said that the variations observed in PQL in this period are due solely to organizational changes, as expressed in other papers [11,19] since, as we have indicated, these changes have only recently transcended normal professional practice.

## Conclusion

PQL is an element that must be considered when planning changes in the health organization to evaluate the impact of these changes in the perception of professionals.

When a health organization undergoes an intentional, episodic change with implications at the macro-management level, improving work control and avoiding an increased workload could maintain PQL perception in workers, despite the magnitude of the organizational change. Though our explanatory model is not designed for confirmation of this, maintenance of professional autonomy, the involvement of professionals in changes and they being made to feel these changes as challenging may be factors that protect against professional burnout, independently of the organizational model.

Moreover, if there are no major variations in salary perceptions, and given an equal workload, the compatibility between job and the rest of social life improves PQL perception.

If one is able to determine the relevance of the changes measured by the PQL-35 instrument, the importance of organizational innovations in PQL can be evaluated with greater precision.

## Competing interests

The author(s) declare that they have no competing interests.

## Authors' contributions

JMF conceived and participated in the design of the study, performed the statistical analysis, was involved in the discussion and the interpretation of the data and draft the manuscript.

TGG was involved in the design of the study, coordinated the last survey and reviewed the interpretation of the results and the final manuscript.

MBL participated in the design of the study, the interpretation of results and reviewed the manuscript.

AAMA helped to discuss the results and review the final manuscript.

JACR conceived the study and coordinated the first two surveys, and reviewed the final manuscript.

## Pre-publication history

The pre-publication history for this paper can be accessed here:



## References

[B1] Weick KE, Quinn RE (1999). Organizational change and development. Annual Review of Psychology.

[B2] Verhaeghe R, Vlerick P, Gemmel P, Van Maele G, De Backer G (2006). Impact of   recurrent changes in the work environment on nurses' psychological   well-being and sickness absence. J Adv Nurs.

[B3] Leese B, Bosanquet N (1996). Changes in general practice organization: survey of general practitioners' views on the 1990 contract and fundholding. Br J Gen Pract.

[B4] Sutherland VJ, Cooper CL (1992). Job stress, satisfaction, and mental health among general practitioners before and after introduction of new contract. BMJ.

[B5] Siegrist J (2000). Place, social exchange and health: proposed sociological framework. Soc Sci Med.

[B6] Karasek R (1989). The political implications of psychosocial work redesign: a model of the psychosocial class structure. Int J Health Serv.

[B7] Van Vegchel N, de Jonge J, Bosma H, Schaufeli W (2005). Reviewing the effort-reward imbalance model: drawing up the balance of 45 empirical studies. Soc Sci Med.

[B8] Johnson JV, Stewart W, Hall EM, Fredlund P, Theorell T (1996). Long-term psychosocial work environment and cardiovascular mortality among Swedish men. Am J Public Health.

[B9] Cabezas Peña C (2000). La calidad de vida de los profesionales. FMC.

[B10] Lichtenstein RL (1984). The job satisfaction and retention of physicians in organized settings: a literature review. Med Care Rev.

[B11] Sibbald B, Bojke Ch, Gravelle H (2003). National survey of job satisfaction and retirement intentions among general practitioners in England. BMJ.

[B12] Grol R, Mokkink H, Smits A, van Eijk J, Beek M, Mesker P, Mesker-Niesten J (1985). Work satisfaction of general practitioners and the quality of patient care. Fam Pract.

[B13] Newman K, Maylor U (2002). The NHS Plan: nurse satisfaction, commitment and retention strategies. Health Serv Manage Res.

[B14] Whitcomb ME, Cohen JJ (2004). The Future of Primary Care Medicine. N Engl J Med.

[B15] LEY 16/2003, de 28 de mayo, de Cohesión y Calidad del Sistema Nacional de Salud. BOE nº 128 de 29 de Mayo de 2003.

[B16] Ley 12/2001, de 21 de diciembre, de Ordenación Sanitaria de la Comunidad de Madrid. BOCM, nº 306 de 26 de Diciembre de 2001.

[B17] Guerra Aguirre J (2001). Las transferencias sanitarias y los médicos de Atención Primaria. Aten Primaria.

[B18] García Ortega C, Almenara Barrios J (2004). Nuevo escenario para el Sistema Nacional de Salud: transferencias y novedades legislativas. Med Clin (Barc).

[B19] Appleton K, House A, Dowell A (1998). A survey of job satisfaction, sources of stress and psychological symptoms among general practitioners in Leeds. Br J Gen Pract.

[B20] Martín J, Cortés JA, Morente M, Caboblanco M, Garijo J, Rodríguez A (2004). Características métricas del Cuestionario de Calidad de Vida Profesional (PQL -35). Gac Sanit.

[B21] Alonso M, Iglesias AI, Franco A (2002). Percepción de la calidad de vida profesional en un área sanitaria de Asturias. Aten Primaria.

[B22] Cortés JA, Martín J, Morente M, Caboblanco M, Garijo J, Rodríguez A (2003). Clima laboral en atención primaria: ¿qué hay que mejorar?. Aten Primaria.

[B23] Muñoz-Seco E, Coll-Benejam JM, Torrent-Quetglas M, Linares-Pou L (2006). Influencia del clima laboral en la satisfacción de los profesionales sanitarios. Aten Primaria.

[B24] Kleinbaum DG, Kupper LL, Muller KE (1988). Applied regression analysis and other multivariate methods.

[B25] Engstrom M, Ljunggren B, Lindqvist R, Carlsson M (2006). Staff satisfaction with work, perceived quality of care and stress in elderly care: psychometric assessments and associations. J Nurs Manag.

[B26] Girard DE, Choi D, Dickey J, Dickerson D, Bloom JD (2006). A comparison study of career satisfaction and emotional states between primary care and speciality residents. Med Educ.

[B27] Sobreques J, Cebria J, Segura J, Rodriguez C, Garcia M, Juncosa S (2003). La satisfacción laboral y el desgaste profesional en los médicos de atención primaria. Aten Primaria.

[B28] Mechanic D (2003). Physician Discontent: Challenges and Opportunities. JAMA.

[B29] Wordsworth S, Skatun D, Scott A, French F (2004). Preferences for general practice jobs: a survey of principals and sessional GPs. Br J Gen Pract.

[B30] French F, Andrew J, Awramenko M, Coutts H, Leighton-Beck L, Mollison J, Needham G, Scott A, Walker K (2005). General practitioner non-principals benefit from flexible working. J Health Organ Manag.

